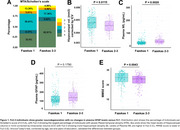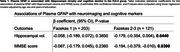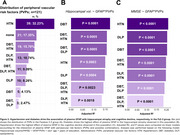# Vascular pathology drives the association of plasma GFAP with gray matter atrophy and cognitive decline in amyloid‐negative individuals

**DOI:** 10.1002/alz70856_105134

**Published:** 2026-01-07

**Authors:** Markley Silva Oliveira, Matheus Scarpatto Rodrigues, Guilherme Povala, Marina Scop Medeiros, João Pedro Ferrari‐Souza, Firoza Z Lussier, Pamela C.L. Ferreira, Guilherme Bauer‐Negrini, Livia Amaral, Andreia Rocha, Sarah Abbas, Hussein Zalzale, Carolina Soares, Pampa Saha, Dana L Tudorascu, Cynthia Felix, Rayan Mroué, Hyun Woong Roh, Eduardo R. Zimmer, Thomas K Karikari, Chang Hyung Hong, Sang Joon Son, Bruna Bellaver, Tharick A Pascoal

**Affiliations:** ^1^ University of Pittsburgh, Pittsburgh, PA, USA; ^2^ Universidade Federal do Rio Grande do Sul, Porto Alegre, RS, Brazil; ^3^ Ajou University School of Medicine, Suwon, Gyeonggido, Korea, Republic of (South); ^4^ Federal University of Rio Grande do Sul (UFRGS), Porto Alegre, RS, Brazil; ^5^ McGill Centre for Studies in Agin, Montreal, QC, Canada; ^6^ Universidade Federal do Rio Grande do Sul, Porto Alegre, Rio Grande do Sul, Brazil; ^7^ Brain Institute of Rio Grande Do Sul, PUCRS, Porto Alegre, RS, Brazil; ^8^ Brain Institute of Rio Grande do Sul, Porto Alegre, Brazil; ^9^ Brain Institute of Rio Grande do Sul (InsCer), PUCRS, Porto Alegre, Rio Grande do Sul, Brazil; ^10^ McGill Centre for Studies in Aging, Montreal, QC, Canada

## Abstract

**Background:**

Amyloid (Aβ) pathology potentiates the association between GFAP, a biomarker for reactive astrogliosis, and neurodegeneration, while it remains unclear whether GFAP is associated with neurodegeneration without Aβ abnormalities. Preclinical studies suggest vascular pathology may activate glial cells, triggering deleterious effects. Here, we tested the hypothesis that, similar to Aβ pathology, vascular pathology may also potentiate the effects of plasma GFAP on neurodegeneration and cognitive decline in Aβ‐negative individuals.

**Method:**

We assessed 324 cognitively impaired (CDR < 0) Aβ‐negative (Centiloid < 24) participants from a memory clinic cohort (BICWALZS), with available CDR global, MMSE, plasma GFAP, clinical assessment of peripheral vascular risk factors [PVPs: hypertension (HTN), diabetes (DBT), and dyslipidemia (DLP)], FLAIR and T1‐based volumetrics. Participants were divided into two groups according to their WMH status: Fazekas 1 (Fz1; n = 203) and Fazekas 2‐3 (Fz2‐3; *n* =  121). Group differences were analyzed using ANCOVA. Associations were assessed using linear regressions, and the contribution of PVPs to the effects of biomarkers was accessed through multicollinearity analysis accounting for age, sex, and years of education.

**Result:**

Individuals in the Fz2‐3 group showed more hippocampal atrophy (Figure 1A, 1B; *p* = 0.0115), plasma NfL levels (1C; *p* = 0.0020), no changes in plasma GFAP levels (Figure 1D) and lower MMSE score (Figure 1E). In the Fz2‐3 group, hippocampal atrophy (Table 1; β: ‐0.179, *p* = 0.0440) and cognitive decline (Table 1; β: ‐0.194, *p* = 0.0390) was associated with plasma GFAP. No abnormalities or associations between biomarkers were found in the Fz1 group. Among the PVPs evaluated, HTN and DBT presented a stronger effect in the association of plasma GFAP in hippocampal atrophy (R^2^: 0.2089; *p* = 0.0001) and cognitive decline (R^2^: 0.1707; *p* < 0.0001), respectively (Figure 3).

**Conclusion:**

Our study found that plasma GFAP is strongly linked to neurodegeneration and MMSE decline in Aβ‐negative individuals with high vascular burden. HTN and DBT, prevalent in the elderly, were the main contributors. This highlights vascular pathology as a key driver of neuroinflammation‐related neurodegeneration, underscoring the importance of managing these conditions to prevent brain atrophy.